# Association between Exposure to Ambient Air Pollution and Rheumatoid Arthritis in Adults

**DOI:** 10.3390/ijerph16071227

**Published:** 2019-04-06

**Authors:** Jiyoung Shin, Jiyoung Lee, Jueun Lee, Eun-Hee Ha

**Affiliations:** 1 Department of Occupational and Environmental Medicine, Ewha Womans University School of Medicine, Seoul 07985, Korea; nellshin5@gmail.com (J.S.); keyma@hanmail.net (J.L.); 2 Department of Medical Science, Ewha Womans University School of Medicine, Seoul 07985, Korea; jueun.claire.lee@gmail.com

**Keywords:** air pollution, rheumatoid arthritis (RA), ozone (O_3_), carbon monoxide (CO), National Health Insurance Data

## Abstract

Environmental factors may play roles in the development of rheumatoid arthritis (RA), and some studies have shown that air pollution was associated with the development of autoimmune disease. This study was designed to investigate the effect of air pollutants on the development of adult RA. A nested case-control cohort study was performed using the National Health Insurance Service-National Sample Cohort during 2002–2014 in Korea. Air pollution data were collected from the National Ambient Air Monitoring System (NAMIS), and exposure levels were extrapolated using geographic information systems. The group with RA (*n* = 444) was compared with a propensity score-matched control group (*n* = 1776), and one-year average concentrations of air pollution were predicted at each patient’s residence. The adjusted binary logistic regression analysis showed a positive association between O_3_ exposure and the incidence risk of RA for the third (odds ratios (OR) = 1.45, 95% confidence intervals (CI): 1.08–1.96) and fourth (OR = 1.35, 95% CI: 1.00–1.83) quartiles in adults over 20 years of age. The third quartile CO exposure was also associated with an increased risk of RA (OR = 1.57, 95% CI: 1.16–2.12). The results of this nationwide population-based study showed that a one-year exposure to CO and O_3_ in adults was associated with an increased risk of RA.

## 1. Introduction

Air pollution is a well-known risk factor that typically affects the cardiovascular and respiratory systems in humans. These effects were demonstrated in both short-term and long-term air pollution studies and result in major adverse public health effects and millions of dollars lost each year [1]. Many cohort studies following exposed individuals have also found associations between air pollution and adverse health outcomes. By reducing air pollution, the burdens of diseases, including heart disease, cancer, and chronic and acute respiratory diseases, can also be reduced [1,2]. In particular, air pollutants, such as particulate matter and ozone, have been suggested to be associated with increased mortality and morbidity and are known to be plausible risk factors for the development of other diseases.

Rheumatoid arthritis (RA) is a chronic disorder of inflammation that targets joints and cartilage and leads to severe disability [3]. Although the pathogenic mechanisms of RA are unclear, several studies have suggested that the risk of developing RA was associated with respiratory exposures to silica, mineral oil, and cigarette smoke, which may activate the immune system, leading to RA [4,5,6].

Furthermore, studies have also suggested that other epidemiological factors, such as noise and traffic-related air pollution, were associated with the development of RA [7]. While exploring the environmental evidence that could trigger RA in a nationwide retrospective cohort study in Taiwan, Chang et al. reported that participants who were exposed to high yearly averages of PM_2.5_ and NO_2_ had an increased risk of RA [8].

Many animal studies have investigated the relationships between different air pollutants and inflammatory cell infiltration, abnormal myocardial mitochondria, and endothelial dysfunction [9,10]. However, the evidence of associations between long-term air pollutant exposure and RA in humans is still lacking. In this study, we aimed to investigate whether exposure to ambient air pollutants (particulate matter with aerodynamic diameters ≤10 µm (PM_10_), nitrogen dioxide (NO_2_), sulfur dioxide (SO_2_), ozone (O_3_), and carbon monoxide (CO)) was associated with an incidence risk of RA.

## 2. Materials and Methods

### 2.1. Data Source and Study Population

The National Health Insurance Service (NHIS) of Korea is a health insurance system with universal coverage. The NHIS established the National Health Information Database, which contains personal information, demographic data, and medical treatment information on Korean citizens that has been collected since its formation in 2000. In 2015, the NHIS released the data of the Korean National Health Insurance Service-National Sample Cohort (NHIS-NSC), which is a representative, population-based cohort proportionally stratified by age, sex, and income levels. From the target population, a representative sample cohort of 1,025,340 participants was randomly selected, comprising 2.2% of the total eligible Korean population in 2002, and followed for 13 years until 2015 or until a participants’ eligibility was disqualified due to death or emigration [11].

**Figure 1:**
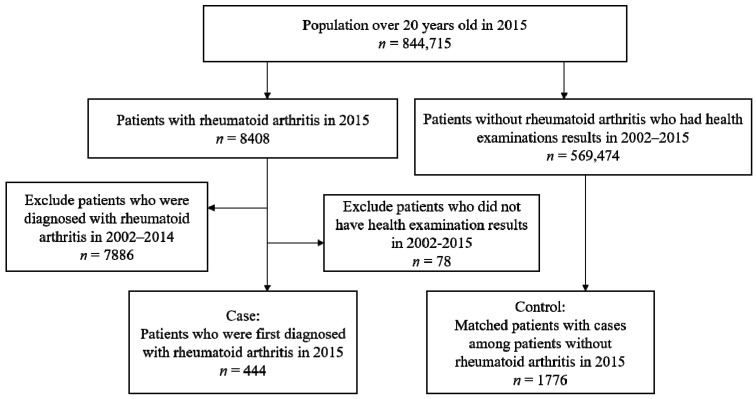
Flow chart of the study population.

We obtained the study population data from the NHIS-NSC between the years 2002 to 2015. From the NHIS-NSC data, we identified 844,715 participants who were over 20 years old in 2015 (Figure 1). We identified the diagnosis data from the patients’ medical treatment based on the International Classification of Diseases, 10th Revision code. Specifically, our main outcome variable was ICD-10 codes corresponding to seropositive rheumatoid arthritis (M05, M050, M053, M058, M059), and the patients who had these ICD-10 codes are considered as RA patients. In this study, we only included patients who had been diagnosed at least once with RA in 2015 (*n* = 8408). In South Korea, the reimbursement criteria for patients with RA between 2009 to 2013 could affect the incidence of RA [12]. Therefore, we excluded the patients who had been diagnosed at least once with RA in 2002–2014 in order to identify recent incident cases of the disease in 2015. Consequently, the remaining population consisted of 7886 subjects.

Of these, we also excluded patients who did not have health examination records (e.g., smoking status and alcohol consumption) or a residential address (*n* = 78). We identified information on patient demographics, including sex (male/female), age group, income level, insurance type, district-level residential address, and medical examination findings, including body mass index (BMI), recorded as underweight (<20.0 kg/m^2^), normal weight (20.0–24.9 kg/m^2^), overweight (25.0–29.9 kg/m^2^), or obesity (≥30 kg/m^2^)), alcohol consumption (rarely/more than 2–3 times per month), smoking status (never/ever), medical treatment, and exercise status. In the case of smoking status, the patient was considered to have ever smoked if they responded in the health examination questionnaire that they smoked in the past or currently smoke.

Ultimately, we identified 444 participants with RA. Then 1:4 propensity score matching was performed, and the propensity scores were calculated based on nearest-neighbor matching without replacement. We selected controls from the NHIS-NSC population who had health examination results and were free of RA. The controls were matched to our identified cases with similar propensity scores by sex, age group in five-year increments, and income level (*n* = 1776). A total of 2220 adults were included in the final analysis. The study protocol was approved by the institutional review board of Ewha Womans University Mokdong Hospital (IRB number: EUMC 2018-07-002).

### 2.2. Estimating Individual Exposure to Air Pollutants

We obtained complete air pollution data (PM_10_, NO_2_, SO_2_, O_3_, and CO) from local district air quality fixed-site monitoring stations managed by the National Ambient Air Monitoring System. Hourly concentrations of these pollutants were measured, and 24-h average concentrations of PM_10_, NO_2_, SO_2_, and CO and 8-h average concentrations of O_3_ were constructed for each measurement site and seventy-five percent of the contributing values were present as the averages were calculated [13].

Because data on air pollution were not available at all sites in South Korea, we applied interpolation techniques using geographic information systems (GIS) tools (ArcGIS Version 9.3, ESRI, Redlands, CA, USA) to estimate air pollution levels in unmonitored areas. All monitoring sites were integrated within the GIS. We interpolated all five air pollutants using ordinary kriging models (a stochastic geostatistical method that takes into account spatial dependence). Kriging is a generic term, adopted by geo-statisticians for a family of generalized least-squares regression algorithms [14]. Kriging calculations based on the surrounding real values and on specified mathematical formulas are optimum data for interpolation if they meet certain conditions (normally distributed and stationary). For this reason, kriging is commonly used for the spatial distribution of air pollution. Until recently, many studies have used kriging for the spatial interpolation of air pollutant distribution [15,16].

We matched the extracted air pollution levels with the participants’ administrative district codes based on the study participants’ residential addresses in the NHIS-NSC database. We also identified changes in individual residential addresses during the study period and matched the different air pollution levels with the participants’ new administrative district codes. Based on the research findings that long-term exposure to moderate levels of air pollution over one year may influence the serum levels of inflammatory markers, we determined the NHIS-NSC participants’ average air pollution exposure during one- to three-year periods between 2012 and 2014 [17].

### 2.3. Statistical Analysis

To estimate the association of air pollutants and RA, we used conditional logistic regression analysis to estimate the association between ambient air pollution exposure (PM_10_, NO_2_, SO_2_, O_3_, and CO), which we assigned to each individual based on the one-year average and RA in adults. We set exposure levels below the 25th percentile for each air pollutant as the reference categories. We also categorized the levels into quartiles with three cutoff points (25th, 50th, and 75th percentiles) for PM_10_ (Quartile 1, <46.26 µg/m^3^; Quartile 2, 46.26–49.05 µg/m^3^; Quartile 3, 49.05–53.62 µg/m^3^; and Quartile 4, ≥53.62 µg/m^3^), NO_2_ (Quartile 1, <20.63 ppb; Quartile 2, 20.63–22.66 ppb; Quartile 3, 22.66–32.47 ppb; and Quartile 4, ≥32.47 ppb), SO_2_ (Quartile 1, <4.72 ppb; Quartile 2, 4.72–5.29 ppb; Quartile 3, 5.29–5.79 ppb; and Quartile 4, ≥5.79 ppb), O_3_ (Quartile 1, <37.66 ppb; Quartile 2, 37.66–39.70 ppb; Quartile 3, 39.70–42.11 ppb; and Quartile 4, ≥42.11 ppb), and CO (Quartile 1, <465.34 ppb; Quartile 2, 465.34–509.70 ppb; Quartile 3, 509.70–552.25 ppb; and Quartile 4, ≥552.25 ppb). For confounding factors, we selected variables that had been identified in the previous literature: insurance type, body mass index (BMI), smoking status (never/ever), alcohol consumption, and exercise status. To disentangle the combined effects of different air pollutants, a multi-pollutant analysis, including all pollutant variables, was considered. We also included two pollutants simultaneously, O_3_ or CO, in the analysis. All analyses were performed using SAS 9.4 software (SAS Institute, Cary, NC, USA), and statistical significance was set at α = 0.05.

## 3. Results

As shown in Figure 1, among the 844,715 individuals who were over 20 years old in 2015, we identified a total of 444 cases based on their ICD-10 codes. We matched 1776 controls who were over 20 years old in 2002–2014, had health examination results, but no rheumatoid arthritis to the group of patients. Table 1 shows the demographic characteristics of the 2220 participants in our study. Of these participants, 69.4% were female and the majority were aged 50–60 years with BMIs in the range of normal weights. Many participants had self-employment insurance, had never smoked tobacco, did not drink alcohol, and exercised less than one time per week.

Table 2 shows that the average air pollution concentration levels varied greatly for each air pollutant. The mean one-year exposure levels for PM_10_, CO, NO_2_, O_3_, and SO_2_ in the case group were 49.41 µg/m^3^, 508.51 ppb, 25.03 ppb, 40.17 ppb, and 5.31 ppb, respectively. The mean exposure levels for PM_10_, CO, NO_2_, O_3_, and SO_2_ in the control group were 49.95 µg/m^3^, 504.75 ppb, 25.73 ppb, 39.84 ppb, and 5.35 ppb, respectively.

Table 3 shows the correlation coefficients for the air pollutant concentration estimates. Spearman’s correlation coefficients of the one-year average of each air pollutant showed positive correlations between PM_10_ and CO (*r* = 0.11), CO and NO_2_ (*r* = 0.73), and NO_2_ and SO_2_ (*r* = 0.29). O_3_ showed negative correlations with all other air pollutants: PM_10_ (*r* = −0.30), CO (*r* = −0.37), NO_2_ (*r* = −0.75), and SO_2_ (*r* = −0.50).

Table 4 shows the odds ratios (OR) and 95% confidence intervals (CI) for the one-year air pollution concentrations and the risk of RA in 2015 among all cases and matched controls. We compared these results based on the lowest quartile of each air pollutant. We also adjusted all the results for insurance type, BMI, smoking status, alcohol consumption, and exercise status. There were no significant associations between PM_10_ and SO_2_ exposure and incidence of RA. As shown in Table 4, 8-h O_3_ exposure was positively associated with RA in the single-pollutant model, and significant associations were observed in the two highest quartiles of O_3_ exposure. For example, we observed the strongest positive effect estimates among all participants between O_3_ and RA (OR = 1.45, 95% CI: 1.08–1.96), as shown in Table 4. However, these results did not show a dose–response relationship between O_3_ exposure and the RA odds. The 24-h CO exposure was also positively associated with RA (OR = 1.57, 95% CI: 1.16–2.12) in the third quartile. In the multi-pollutant model, which included all air pollutant variables, the effect estimate of O_3_ was attenuated. However, the effect estimate of CO became larger and showed a dose–response relationship. We observed inverse associations for 24-h NO_2_ exposure and RA. Exposure levels above the fourth quartile for NO_2_ were associated with decreases in the effect estimates, which were evident in the single-pollutant model.

We also examined the association between one- to three-year average air pollution concentrations and the risk of RA in 2015 among all cases and matched controls. In the two-year exposure model, CO and O_3_ exposure were positively related with an increased risk of RA. However, in the three-year exposure model, only CO exposure correlated with an increased risk of RA (Supplementary Table S1).

**Figure 2:**
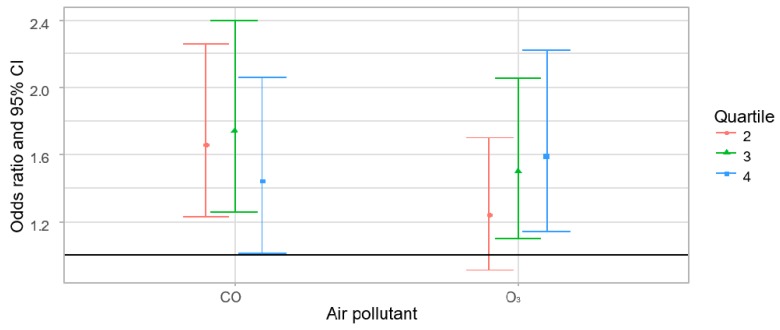
Conditional logistic regression results from the two-pollutant models for adjusted associations between one-year CO and O_3_ concentrations and rheumatoid arthritis in adults ^abc^. ^a^ Results adjusted for insurance type, body mass index (BMI), smoking status, alcohol consumption, and exercise status; ^b^ Results compared with the exposure in the lowest quartile (first quartile); ^c^ Two-pollutant model: O_3_ + CO.

Figure 2 shows the two-pollutant models used to identify the combined effects. In the two-pollutant models for adjusted associations between the one-year CO and O_3_ concentrations and RA in adults, both CO and O_3_ still showed positive associations with the incidence of RA. The positive effects of CO in the second quartile (OR = 1.66, 95% CI: 1.23–2.26), the third quartile (OR = 1.74, 95% CI: 1.26–2.40), and the fourth quartile (OR = 1.44, 95% CI: 1.01–2.06), and of O_3_ in the second quartile (OR = 1.24, 95% CI: 0.91–1.70), the third quartile (OR = 1.50, 95% CI: 1.10–2.05), and the fourth quartile (OR = 1.59, 95% CI: 1.14–2.22) were stronger than the effects in the single-pollutant model. The two- to three-year two-pollutant models also showed a significant relationship between CO and incidence of RA (Supplementary Table S2).

## 4. Discussion

Through the use of a large, national 12-year cohort database in South Korea, we conducted a nested case-control study and were able to efficiently evaluate the effects of individual-level estimates of air pollutants on the risk of RA. We found that ambient exposure to CO and O_3_ was associated with an increased risk of RA in adults. In the one-year air pollution exposure model, when we compared subjects who had been exposed to the lowest quartile of O_3_ exposure, the risk of RA increased significantly in the highest quartile of one-year ozone exposure by approximately 1.35-fold. Furthermore, when we used a two-pollutant model for CO and O_3_, the effect became larger than the single-pollutant model. However, we also noted negative associations for NO_2_ in the quartile models but no consistent observation for PM_10_ and SO_2_

Multi-pollutant models were built to assess the association between combined exposure to multiple air pollutants and the odds of developing RA. Although multi-pollutant models can induce bias and variance inflation in the analyses if air pollutant variables are highly correlated, it was unclear which model showed less biased results [18,19]. Therefore, we presented all single-, two-, and multi-pollutant model results.

We found positive relationships between exposure to some gaseous pollutants (CO and O_3_) and the risk of RA. Although we found no dose–response relationship between CO and O_3_ exposure and RA in the single-pollutant model, CO showed a dose–response relationship with RA in the multi-pollutant model. Some previous research has shown relatively steep nonlinear exposure–response curves at low doses that leveled off at higher exposures. [20,21] In air pollution exposure, some studies on non-linear air pollution exposure have suggested, that depending on the agent, nonlinear models may have plausibility as well [22,23,24]. However, to the best of our knowledge, no studies have shown a dose–response association between CO or O_3_ exposure and the risk of RA. Further research is needed to elucidate the biological mechanisms underlying this nonlinear response.

Although we could not find dose–response patterns by exposure level quartile, O_3_ showed dose–response patterns in the two-pollutant model for both CO and O_3_. The risk of RA was increased even more (1.59-fold) in the highest quartile of O_3_ exposure.

In this study, we also found a negative relationship between NO_2_ exposure and the incident risk of RA. NO_2_ was negatively associated with RA for the highest quartiles of exposure in both single-pollutant models. This observed relationship may be explained by the chemical coupling bond between ambient O_3_ and NO_2_. The O_3_ and NO_2_ exposure levels were inextricably linked; lower NO_2_ exposure invariably occurred with higher O_3_ exposure. That is, individuals who were often exposed to high O_3_ levels were also exposed to low levels of NO_2_ [25].

Our findings are largely consistent with those from other previous studies on air pollution and RA. For example, another study using the population-based Border Air Quality Study cohort in Canada reported that ground-level O_3_ was associated with 15% and 26% increased risks of RA in cases identified by the RA-ICD-9 code and by RA-prescriptions, respectively [7]. The authors of that study also found 29% and 56% increased risks with ground-level O_3_ levels in the highest versus the lowest quintiles, respectively. Furthermore, in a nationwide retrospective cohort study in Taiwan, researchers found that there was no significant association between PM_10_ exposure and RA, and the association remained stable after adjustment for potential confounding factors [8]. In another previous study by Hart et al., the authors did not observe any risk increase with PM_2.5_, PM_10_, NO_2_, or SO_2_, providing evidence against exposure to those air pollutants as a risk factor for RA [26]. However, a Swedish case-control study suggested an increased risk of RA incidence in the Stockholm area with increases in NO_2_ and SO_2_, and stronger associations with the antibody to citrullinated protein antigens (ACPA)-negative phenotype [27].

In this study, we used a representative, population-based cohort from the NHIS-NSC, which has a major strength in that its applicability in research is ensured. The data are stable and extensive because the NHIS-NSC is constructed based on a robust sampling design generated by the government that allows for adequate statistical power [11]. However, since our study only considered patients who received a diagnosis of seropositive rheumatoid arthritis by ICD-10 code, further studies, such as research studies considering air pollution and RA status according to the presence of antibodies, are needed.

We assigned individual exposure levels for air pollutants by kriging using GIS. Diverse methodologies, including statistical interpolation, line dispersion models, land use regression models, and hybrid models have been suggested to estimate air pollution levels in order to minimize exposure misclassifications at unmonitored locations, which is a key challenge in epidemiological studies designed to find associations between air pollution and health outcomes [28]. We performed a statistical interpolation method (i.e., kriging) in our study because the air pollutant data was collected from air pollution monitoring stations at specific sites located across Korea during the study period. Kriging estimates an unknown value as a weighted average from the nearest sampling points and is an advanced geostatistical procedure that takes spatial dependence into account. However, our air pollutant data were collected from designated nationwide monitoring sites and these nationwide sites are often installed to monitor certain areas affected by heavy traffic and industrial emissions. Therefore, interpolating these monitoring data could have overestimated the true air pollution levels.

## 5. Conclusions

In conclusion, the present study in Korea suggested an increased risk of incident RA in adults exposed to CO and O_3_. Our study extended the prior results of research regarding the effect of air pollution on the risk of RA. With substantial concerns regarding air pollution-induced adverse health outcomes, much effort has been made to ameliorate air pollution levels, however, the precise range in which human exposure to air pollution is safe is still unknown. Therefore, it is necessary to evaluate changes in different air pollutant concentrations, as well as the degree of health damage. Although additional studies are necessary, the findings of the current study can serve as guidelines for implementing air quality policies for environmental concerns and social welfare.

## Figures and Tables

**Table 1:** Descriptive characteristics of the study participants (*n* = 2220) in 2015.

Characteristics	All	
	Cases *n* = 444	Controls *n* = 1776
Sex		
Male	136 (30.6%)	544 (30.6%)
Female	308 (69.4%)	1232 (69.4%)
Age		
20–30	6 (1.4%)	24 (1.4%)
30–40	33 (7.4%)	132 (7.4%)
40–50	86 (19.4%)	344 (19.4%)
50–60	147 (33.1%)	588 (33.1%)
60–70	100 (22.5%)	400 (22.5%)
70–80	55 (12.4%)	220 (12.4%)
≥80	17 (3.8%)	68 (3.83%)
Household income relative to the median (%)		
Lowest (<20%)	91 (20.5%)	364 (20.5%)
20–80%	171 (38.5%)	684 (38.5%)
Highest (>80%)	182 (41.0%)	728 (41.0%)
BMI		
Underweight	44 (9.9%)	196 (11.0%)
Normal weight	261 (58.8%)	993 (55.9%)
Overweight	120 (27.0%)	511 (28.8%)
Obesity	19 (4.3%)	76 (4.3%)
Insurance type		
Employee insured	102 (23.0%)	481 (27.1%)
Self-employed insured	328 (73.9%)	1262 (71.1%)
Medical-aid beneficiary	14 (3.2%)	33 (1.86%)
Smoking status		
Never	336 (75.7%)	1335 (75.3%)
Ever	108 (24.3%)	439 (24.8%)
Alcohol consumption		
Rarely	317 (71.4%)	1172 (66.1%)
Over 2 or 3 times per month	127 (28.6%)	601 (33.9%)
Exercise status		
<1 time per week	256 (57.7%)	983 (55.4%)
≥1 time per week	188 (42.3%)	790 (44.6%)

BMI: body mass index.

**Table 2:** Distribution of air pollution exposure concentrations during a one-year period.

Air Pollutant		Air Pollutant Distribution					
		Minimum	25th	50th (Median)	75th	Maximum	Mean ± SD
Total *n* = 2220	PM_10_ µg/m^3^	40.04	46.26	49.05	53.62	62.49	49.85 ± 5.33
	CO (ppb)	351.83	465.34	509.70	552.25	643.10	505.50 ± 58.63
	NO_2_ (ppb)	15.68	20.63	22.66	32.47	36.09	25.59 ± 6.04
	O_3_ (ppb)	34.61	37.66	39.70	42.11	45.67	39.90 ± 2.75
	SO_2_ (ppb)	4.22	4.72	5.29	5.79	8.52	5.34 ± 0.79
Case *n* = 444	PM_10_ µg/m^3^	40.04	45.38	48.14	53.57	62.49	49.41 ± 5.50
	CO (ppb)	351.83	471.50	510.61	546.05	643.10	508.51 ± 53.10
	NO_2_ (ppb)	15.68	20.61	22.34	32.02	36.09	25.03 ± 5.87
	O_3_ (ppb)	34.61	37.95	40.15	42.17	45.67	40.17 ± 2.67
	SO_2_ (ppb)	4.22	4.58	5.17	5.73	8.52	5.31 ± 0.87
Control *n* = 1776	PM_10_ µg/m^3^	40.04	46.39	49.26	53.62	62.49	49.95 ± 5.28
	CO (ppb)	351.83	464.51	509.70	552.87	643.10	504.75 ± 59.92
	NO_2_ (ppb)	16.11	20.63	22.81	32.50	36.09	25.73 ± 6.07
	O_3_ (ppb)	34.61	37.59	39.68	42.10	45.67	39.84 ± 2.77
	SO_2_ (ppb)	4.22	4.78	5.29	5.79	8.52	5.35 ± 0.77

SD: standard deviation.

**Table 3:** Spearman correlation coefficients for the estimates of air pollutant concentrations.

Air Pollutant		1-Year Average Concentration				
		PM_10_	CO	NO_2_	O_3_	SO_2_
1-Year Average Concentration	PM_10_	1	0.11 *	0.08 *	−0.30 *	0.29 *
	CO		1	0.73 *	−0.37 *	−0.04
	NO_2_			1	−0.75 *	0.29 *
	O_3_				1	−0.50 *
	SO_2_					1

* Coefficients were statistically significant (*p* < 0.05).

**Table 4:** Conditional logistic regression results from the single- and multi-pollutant models for adjusted associations between one-year air pollutant concentration and rheumatoid arthritis in adults ^a^ (*n* = 2220).

Air Pollutant	Single-Pollutant Models		Multi-Pollutant Models ^c^	
	Quartile ^b^	OR (95% CI)	Quartile ^b^	OR (95% CI)
O_3_ (ppb)	2	1.17 (0.86–1.59)	2	1.13 (0.78–1.63)
	3	1.45 (1.08–1.96)	3	1.21 (0.77–1.91)
	4	1.35 (1.00–1.83)	4	1.21 (0.69–2.13)
PM_10_ µg/m^3^	2	0.87 (0.65–1.16)	2	0.98 (0.69–1.41)
	3	0.79 (0.59–1.07)	3	0.85 (0.60–1.22)
	4	0.85 (0.64–1.13)	4	0.82 (0.57–1.19)
CO (ppb)	2	1.52 (1.12–2.04)	2	1.74 (1.24–2.44)
	3	1.57 (1.16–2.12)	3	1.83 (1.24–2.70)
	4	1.15 (0.83–1.58)	4	1.83 (1.11–3.01)
NO_2_ (ppb)	2	0.96 (0.71–1.28)	2	1.03 (0.73–1.46)
	3	1.00 (0.75–1.33)	3	0.99 (0.64–1.52)
	4	0.72 (0.53–0.98)	4	0.71 (0.37–1.36)
SO_2_ (ppb)	2	0.79 (0.60–1.06)	2	0.86 (0.58–1.28)
	3	0.75 (0.56–1.00)	3	0.86 (0.55–1.33)
	4	0.76 (0.57–1.02)	4	0.98 (0.62–1.55)

**^a^** Results adjusted for insurance type, body mass index (BMI), smoking status, alcohol consumption, and exercise status; **^b^** Results compared with the exposure in the lowest quartile (first quartile); **^c^** Multi-pollutant model: O_3_ + PM_10_ + CO + NO_2_ + SO_2_.

## Supplementary Materials

The following are available online at https://www.mdpi.com/1660-4601/16/7/1227/s1, Table S1: Conditional logistic regression results from the single- and multi-pollutant models for adjusted associations between two- to three-year air pollutant concentrations and rheumatoid arthritis in adults ^a^ (*n* = 2220). Table S2: Conditional logistic regression results from the two-pollutant models for adjusted associations between one-year CO and O_3_ concentrations and rheumatoid arthritis in adults ^abc^ (*n* = 2220).

## References

[1] Brunekreef B., Holgate S.T. (2002). Air Pollution and Health. Lancet.

[2] WHO (2018). Ambient (Outdoor) Air Quality and Health. 2018.

[3] Lawrence R.C., Helmick C.G., Arnett F.C., Deyo R.A., Felson D.T., Giannini E.H., Heyse S.P., Hirsch R., Hochberg M.C., Hunder G.G. (1998). Estimates of the Prevalence of Arthritis and Selected Musculoskeletal Disorders in the United States. Arthritis Rheum. Off. J. Am. Coll. Rheumatol..

[4] Stolt P., Kallberg H., Lundberg I., Sjogren B., Klareskog L., Alfredsson L., EIRA Study Group (2005). Silica Exposure is Associated with Increased Risk of Developing Rheumatoid Arthritis: Results from the Swedish EIRA Study. Ann. Rheum. Dis..

[5] Sverdrup B., Källberg H., Bengtsson C., Lundberg I., Padyukov L., Alfredsson L., Klareskog L. (2005). Association between Occupational Exposure to Mineral Oil and Rheumatoid Arthritis: Results from the Swedish EIRA Case–control Study. Arthritis Res. Ther..

[6] Criswell L.A., Merlino L.A., Cerhan J.R., Mikuls T.R., Mudano A.S., Burma M., Folsom A.R., Saag K.G. (2002). Cigarette Smoking and the Risk of Rheumatoid Arthritis among Postmenopausal Women: Results from the Iowa Women’s Health Study. Am. J. Med..

[7] De Roos A.J., Koehoorn M., Tamburic L., Davies H.W., Brauer M. (2014). Proximity to Traffic, Ambient Air Pollution, and Community Noise in Relation to Incident Rheumatoid Arthritis. Environ. Health Perspect..

[8] Chang K., Hsu C., Muo C., Hsu C.Y., Liu H., Kao C., Chen C., Chang M., Hsu Y. (2016). Air Pollution Exposure Increases the Risk of Rheumatoid Arthritis: A Longitudinal and Nationwide Study. Environ. Int..

[9] Kodavanti U.P., Thomas R., Ledbetter A.D., Schladweiler M.C., Shannahan J.H., Wallenborn J.G., Lund A.K., Campen M.J., Butler E.O., Gottipolu R.R. (2011). Vascular and Cardiac Impairments in Rats Inhaling Ozone and Diesel Exhaust Particles. Environ. Health Perspect..

[10] Tamagawa E., Bai N., Morimoto K., Gray C., Mui T., Yatera K., Zhang X., Xing L., Li Y., Laher I. (2008). Particulate Matter Exposure Induces Persistent Lung Inflammation and Endothelial Dysfunction. Am. J. Physiol.-Lung Cell. Mol. Physiol..

[11] Lee J., Lee J.S., Park S., Shin S.A., Kim K. (2016). Cohort Profile: The National Health Insurance Service—National Sample Cohort (NHIS-NSC), South Korea. Int. J. Epidemiol..

[12] Lee M., Shin J., Park S., Kim D., Cha H., Lee E. (2018). Persistence of Biologic Disease-Modifying Antirheumatic Drugs in Patients with Rheumatoid Arthritis: An Analysis of the South Korean National Health Insurance Database. Semin. Arthritis Rheum..

[13] Korea Environment Corporation (2018). Air Korea.

[14] Diodato N. (2005). The Influence of Topographic Co-variables on the Spatial Variability of Precipitation Over Small Regions of Complex Terrain. Int. J. Climatol..

[15] Montero J., Fernández-Avilés G. (2018). Functional Kriging Prediction of Atmospheric Particulate Matter Concentrations in Madrid, Spain: Is the New Monitoring System Masking Potential Public Health Problems?. J. Clean. Prod..

[16] Chen L., Gao S., Zhang H., Sun Y., Ma Z., Vedal S., Mao J., Bai Z. (2018). Spatiotemporal Modeling of PM 2.5 Concentrations at the National Scale Combining Land use Regression and Bayesian Maximum Entropy in China. Environ. Int..

[17] Panasevich S., Leander K., Rosenlund M., Ljungman P., Bellander T., de Faire U., Pershagen G., Nyberg F. (2009). Associations of Long- and Short-Term Air Pollution Exposure with Markers of Inflammation and Coagulation in a Population Sample. Occup. Environ. Med..

[18] Rothman K.J., Greenland S., Lash T.L. (2008). Modern Epidemiology.

[19] Schisterman E.F., Cole S.R., Platt R.W. (2009). Overadjustment Bias and Unnecessary Adjustment in Epidemiologic Studies. Epidemiology.

[20] Hertz-Picciotto I., Smith A.H. (1993). Observations on the Dose-Response Curve for Arsenic Exposure and Lung Cancer. Scand. J. Work Environ. Health.

[21] Vineis P., Kogevinas M., Simonato L., Brennan P., Boffetta P. (2000). Levelling-Off of the Risk of Lung and Bladder Cancer in Heavy Smokers: An Analysis Based on Multicentric Case-Control Studies and a Metabolic Interpretation. Mutat. Res..

[22] Xie W., Li G., Zhao D., Xie X., Wei Z., Wang W., Wang M., Li G., Liu W., Sun J. (2015). Relationship between Fine Particulate Air Pollution and Ischaemic Heart Disease Morbidity and Mortality. Heart.

[23] Smith R.L., Spitzner D., Kim Y., Fuentes M. (2000). Threshold Dependence of Mortality Effects for Fine and Coarse Particles in Phoenix, Arizona. J. Air Waste Manag. Assoc..

[24] Daniels M.J., Dominici F., Samet J.M., Zeger S.L. (2000). Estimating Particulate Matter-Mortality Dose-Response Curves and Threshold Levels: An Analysis of Daily Time-Series for the 20 Largest US Cities. Am. J. Epidemiol..

[25] Clapp L.J., Jenkin M.E. (2001). Analysis of the Relationship between Ambient Levels of O3, NO2 and NO as a Function of NOx in the UK. Atmos. Environ..

[26] Hart J.E., Källberg H., Laden F., Costenbader K.H., Yanosky J.D., Klareskog L., Alfredsson L., Karlson E.W. (2013). Ambient Air Pollution Exposures and Risk of Rheumatoid Arthritis. Arthritis Care Res..

[27] Hart J.E., Kallberg H., Laden F., Bellander T., Costenbader K.H., Holmqvist M., Klareskog L., Alfredsson L., Karlson E.W. (2013). Ambient Air Pollution Exposures and Risk of Rheumatoid Arthritis: Results from the Swedish EIRA Case-Control Study. Ann. Rheum. Dis..

[28] Jerrett M., Burnett R.T., Ma R., Pope C.A., Krewski D., Newbold K.B., Thurston G., Shi Y., Finkelstein N., Calle E.E. (2005). Spatial Analysis of Air Pollution and Mortality in Los Angeles. Epidemiology.

