# Supplementary material for: Association between Exposure to Ambient Air Pollution and Rheumatoid Arthritis in Adults
Source: Int J Environ Res Public Health. 2019 Apr 6;16(7):1227. doi: 10.3390/ijerph16071227 (PMC6480037; doi:10.3390/ijerph16071227)
Supplement: Supplementary file 1 [file ijerph-16-01227-s001.pdf]

Table S1. Conditional logistic regression results from the single- and multi-pollutant models for adjusted associations between two- to three-year air pollutant concentrations and rheumatoid arthritis in adults [a] (n = 2220).

	Single-Pollutant Models		Multi-Pollutant Models	
**Air Pollutant**	**Quartile [b]**	**OR (95% CI)**	**Quartile [b]**	**OR (95% CI)**
**2-Year Concentration**				
O_3 (ppb)	2	0.91 (0.66–1.24)	2	0.75 (0.48–1.18)
	3	1.48 (1.10–1.99)	3	1.09 (0.65–1.81)
	4	1.18 (0.87–1.59)	4	0.82 (0.45–1.49)
PM_{10} (μg/m^3)	2	0.79 (0.59–1.06)	2	0.89 (0.63–1.27)
	3	0.72 (0.54–0.97)	3	0.80 (0.54–1.19)
	4	0.83 (0.63–1.11)	4	0.90 (0.59–1.37)
CO (ppb)	2	1.41 (1.05–1.90)	2	1.61 (1.15–2.25)
	3	1.37 (1.01–1.86)	3	1.53 (1.01–2.31)
	4	1.13 (0.82–1.54)	4	1.49 (0.90–2.47)
NO_2 (ppb)	2	0.97 (0.73–1.30)	2	0.90 (0.62–1.31)
	3	1.04 (0.78–1.39)	3	0.93 (0.57–1.51)
	4	0.76 (0.56–1.03)	4	0.71 (0.36–1.37)
SO_2 (ppb)	2	0.64 (0.47–0.85)	2	0.65 (0.45–0.94)
	3	0.69 (0.52–0.91)	3	0.74 (0.48–1.14)
	4	0.72 (0.54–0.96)	4	0.81 (0.55–1.19)
**3-Year Concentration**				
O_3 (ppb)	2	0.88 (0.64–1.20)	2	0.64 (0.41–1.00)
	3	1.24 (0.92–1.67)	3	0.78 (0.48–1.26)
	4	1.18 (0.88–1.58)	4	0.65 (0.37–1.15)
PM_{10} (μg/m^3)	2	0.73 (0.55–0.99)	2	0.84 (0.60–1.18)
	3	0.84 (0.63–1.13)	3	1.02 (0.70–1.48)
	4	0.84 (0.63–1.11)	4	0.99 (0.68–1.44)
CO (ppb)	2	1.42 (1.06–1.91)	2	1.49 (1.08–2.05)
	3	1.33 (0.98–1.80)	3	1.67 (1.06–2.63)
	4	1.1 (0.80–1.51)	4	1.63 (0.94–2.84)
NO_2 (ppb)	2	0.94 (0.70–1.25)	2	0.90 (0.60–1.37)
	3	0.98 (0.74–1.30)	3	0.74 (0.42–1.30)
	4	0.71 (0.52–0.96)	4	0.47 (0.22–0.97)
SO_2 (ppb)	2	0.66 (0.50–0.89)	2	0.71 (0.48–1.04)
	3	0.66 (0.50–0.88)	3	0.72 (0.47–1.09)
	4	0.70 (0.53–0.94)	4	0.79 (0.52–1.19)

[a] Results adjusted for insurance type, body mass index (BMI), smoking status, alcohol consumption, and exercise status; [b] Results compared with the exposure in the lowest quartile (first quartile).

Table S2. Conditional logistic regression results from the two-pollutant models for adjusted associations between one-year CO and O_3 concentrations and rheumatoid arthritis in adults [abc] (n = 2220).

**2-Year Concentration**	**Quartile [b]**	**OR (95% CI)**
O_3	2	0.93 (0.68–1.29)
	3	1.57 (1.15–2.14)
	4	1.45 (1.01–2.07)
CO	2	1.59 (1.16–2.18)
	3	1.60 (1.14–2.25)
	4	1.42 (0.98–2.04)
**3-Year Concentration**		
O_3	2	0.92 (0.66–1.28)
	3	1.33 (0.95–1.86)
	4	1.41 (0.97–2.04)
CO	2	1.49 (1.10–2.01)
	3	1.59 (1.13–2.26)
	4	1.37 (0.94–2.00)

[a] Results adjusted for insurance type, body mass index (BMI), smoking status, alcohol consumption, and exercise status; [b] Results compared with the exposure in the lowest quartile (first quartile); [c] Two-pollutant model: CO + O_3.
